# Impact of epiphora on vision-related quality of life

**DOI:** 10.1186/1471-2415-15-6

**Published:** 2015-01-21

**Authors:** Jae-Ho Shin, Yoon-Duck Kim, Kyung In Woo, Korean Society of Ophthalmic Plastic and Reconstructive Surgery (KSOPRS)

**Affiliations:** Department of Ophthalmology, Kyung Hee University Hospital at Gangdong, Kyung Hee University College of Medicine, Seoul, South Korea; Department of Ophthalmology, Samsung Medical Center, Sungkyunkwan University School of Medicine, #81 Irwon-ro, Gangnam-gu, 135-710 Seoul, South Korea

**Keywords:** Dacryocystorhinostomy, Epiphora, Self-assessment questionnaire, Vision-related quality of life

## Abstract

**Background:**

The study aimed to evaluate vision-related quality of life (QOL) in epiphora (excessive tear production) patients.

**Methods:**

A total of 342 epiphora patients who visited ophthalmology clinics at 21 general hospitals during a 1-week period were enrolled; 245 females and 97 males with a mean age of 52 ± 13 years. Participants completed a self-administered questionnaire on the extent to which epiphora limited their daily activities. Questions on frequency of discomfort with daily activities were answered on scale of a 0–4. Daily activities that were assessed included reading, daytime and nighttime driving, working at a computer, watching TV, work-related activities, household activities, outdoor activities, interpersonal relations, and general happiness. A correlation analysis was performed between the scores and patient ages. Scores were compared with the clinical factors of gender, bilaterality, and lacrimal irrigation pattern. Presurgical and postsurgical scores in a subset of epiphora patients who underwent surgery were compared.

**Results:**

Outdoor activities were among those that epiphora most significantly hindered. Age had a negative correlation with interpersonal relations scores. Female patients tended to have more discomfort than males in conducting household activities, outdoor activities, and interpersonal relations. Bilaterality showed no differences in QOL. Patients with complete obstruction of lacrimal irrigation recorded higher scores in all daily activities than those with partial or no obstruction. For vision-related QOL, post-surgical scores were improved significantly compared with pre-surgical scores.

**Conclusion:**

Epiphora can affect a broad array of daily activities. Corrective measures for epiphora can improve vision-related QOL, and this may provide guidance for physicians in managing epiphora patients.

## Background

A stable tear film is vital for maintaining optical quality and normal functioning of the eye. Symptomatic epiphora (excessive tear production) can occur if secreted tears do not drain properly. Epiphora is a commonly reported symptom and has even been described on ancient Egyptian papyrus artifacts and in the era of Hippocrates [1]. Despite a long history of recognition of epiphora, there are few reports on its impact on patients’ daily activities and social lives.

Woog reported the average annual incidence of symptomatic acquired lacrimal outflow obstruction was revealed as 30.47 per 100,000, and the incidence increased with age [2]. As life expectancy increases, according to demographic trends in many countries, the epiphora prevalence will continue to increase and significantly affect quality of life (QOL). Kafil-Hussain et al. suggested that patients with epiphora suffer the same, if not greater visual handicap than patients awaiting a second cataract surgery [3].

Epiphora’s impact among certain patient group’s daily and social lives has been discussed. QOL assessment studies have mostly been performed using the Glasgow Benefit Inventory (GBI) for specific lacrimal procedures [4–6]. Smirnov et al. developed a specific Nasolacrimal Duct Obstruction Symptom Score questionnaire to evaluate lacrimal surgery outcomes and showed that the score correlated with the GBI and, compared with GBI alone, gave more information about benefits after endonasal dacryocystorhinostomy [7].

We therefore aimed to assess epiphora’s influence on vision related QOL by using a self-assessment questionnaire, through which we established and evaluated correlations of clinical factors to the QOL scores.

## Methods

This study targeted epiphora patients who visited the ophthalmologic clinics at 21 general hospitals in South Korea. The study’s purpose was explained to patients and written consent was obtained from participants. Data gathering was carried out after ethical review by the Samsung Medical Center [Board of Clinical Research Ethics].

The study was performed during the week of October 12–17, 2009. The inclusion criterion was symptomatic epiphora from tear drainage abnormality. All patients older than 18 years were informed of the study and those who agreed to respond to questionnaires were included. We excluded those who could not independently answer the questionnaire and/or had other ocular diseases that could significantly affect visual acuity.

The questionnaire consisted of 10 items concerning common vision-related symptoms that affect performing of daily activities based on the Ocular Surface Diseased Index (OSDI). The questionnaire included reading, daytime and nighttime driving, working at a computer, watching TV, work-related activities, household activities, outdoor activities, interpersonal relations, and general happiness. Frequency of discomfort for daily activities was assessed on a scale of 0 (never) to 4 (always). Questions to which patients did not respond were excluded from statistical analysis. After an initial explanation of the questionnaire, the participants filled it out by themselves. Ophthalmologic examination was performed, including measurement of tear meniscus height using variable beam heights on a slit lamp, and lacrimal irrigation.

A total of 342 patients were included in the study with an average age of 52 ± 13 years (range: 18–89 years); 97 males and 245 females were included (Table 1). Of the total, 35 were followed-up on after dacryocystorhinostomy and were asked about the degree of discomfort from lacrimation in daily life, comparing presurgery and postsurgery values.

**Table 1:** **Characteristics of epiphora patients in this survey**

Patients enrolled in the study	
Age (mean ± S.D [range], years)	52 ± 13 (18–89)
Male/Female	97/245
Unilateral/Bilateral	165/115
Increased (>0.2 mm)/Low-to-normal tear meniscus height (≤0.2 mm)	236/40
Good-or-partial passage/No passage on lacrimal irrigation	100/176

The relationships between the self-assessment score for each question and the clinical features including age, gender, bilaterality, and the result of lacrimal syringing were analyzed. Program R (version 2.11.1) was used for statistical analyses. A Mann–Whitney test was used when comparisons of two groups were needed. Spearman’s rank-order correlation was used for continuous variable analysis such as with age. A signed rank test was used for comparing presurgery and postsurgery self-assessment scores. P-values < 0.05 were considered statistically significant.

This study abided with the tenets of the Declaration of Helsinki.

## Results

Epiphora significantly hindered outdoor activities (mean response: 2.80; Table 2). In the analysis on age, interpersonal relations showed a negative correlation (P = 0.048, Table 3), which reflected younger patients feeling greater discomfort, with statistical significance. Analyzing the gender effect on daily discomfort in daily life, females had higher scores for household activities, outdoor activities, and interpersonal relations than males (Table 4). One-sided and two-sided epiphora patients showed no significant differences in scores. Patients with complete obstruction of lacrimal irrigation showed higher symptom scores than those who had patent lacrimal systems for every activity, except for daytime and nighttime driving (Table 5).

**Figure 1:**
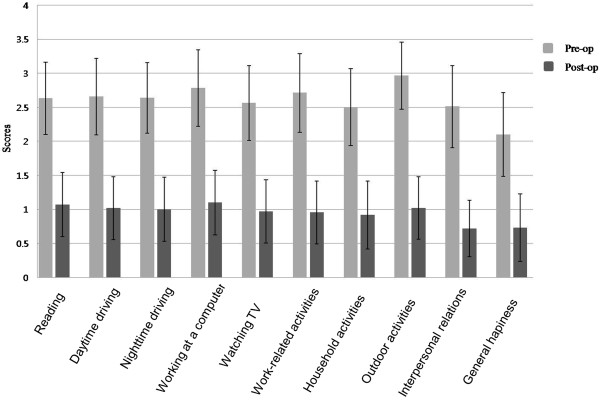
**Scores before and after dacryocystorhinostomy (mean, S.D.).** All scores were significantly decreased after surgery (P < 0.01).

Self-assessed scores significantly improved after dacryocystorhinostomy postsurgery compared with before dacryocystorhinostomy. Work-related activities showed the largest differences in the 10 questions, followed by outside activities, working at a computer, and interpersonal relations (mean difference: 1.83; P < 0.01; Figure 1).

**Table 2:** **Averaged symptom scores of the epiphora patients (mean ± S.D.)**

Item	Symptom score	No. of respondents
Reading	2.45 ± 1.22	329
Daytime driving	2.20 ± 1.24	174
Nighttime driving	2.30 ± 1.29	165
Working at a computer	2.49 ± 1.18	200
Watching TV	2.25 ± 1.19	342
Work-related activities	2.2 7 ± 1.26	215
Household activities	2.04 ± 1.23	322
Outdoor activities	2.80 ± 1.14	342
Interpersonal relations	2.40 ± 1.24	342
General happiness	1.91 ± 1.32	317

**Table 3:** **Correlation of self-administered questionnaire scores with age**

Item	Spearman’s rank correlation	P value
Reading	0.12	0.121
Daytime driving	−0.02	0.818
Nighttime driving	0.04	0.725
Working at a Computer	−0.08	0.364
Watching TV	−0.02	0.756
Work-related activities	−0.07	0.483
Household activities	0.05	0.491
Outdoor activities	−0.07	0.325
Interpersonal relations	−0.14	0.048^*^
General happiness	−0.01	0.906

^*^P < 0.05.

**Table 4:** **Comparison of self-administered questionnaire scores by gender**

Item	Group	Mean	P value
Reading	Females (n = 126)	2.51	0.404
	Males (n = 55)	2.33	
Daytime driving	Females (n = 56)	2.23	0.831
	Males (n = 43)	2.16	
Nighttime driving	Females (n = 51)	2.39	0.541
	Males (n = 43)	2.19	
Working at a Computer	Females (n = 71)	2.46	0.730
	Males (n = 38)	2.53	
Watching TV	Females (n = 139)	2.30	0.332
	Males (n = 56)	2.13	
Work-related activities	Females (n = 67)	2.30	0.764
	Males (n = 46)	2.24	
Household activities	Females (n = 142)	2.18	0.006^*^
	Males (n = 40)	1.58	
Outdoor activities	Females (n = 139)	2.90	0.047^*^
	Males (n = 55)	2.56	
Interpersonal relations	Females (n = 140)	2.54	0.017^*^
	Males (n = 57)	2.07	
General happiness	Females (n = 126)	1.99	0.200
	Males (n = 44)	1.68	

^*^P < 0.05.

**Table 5:** **Comparison of self-administered questionnaire results by results of a lacrimal irrigation test**

Item	Group	Mean	P value
Reading	Completely obstructed in at least one eye (n = 116)	2.65	0.008^*^
	Completely or partially patent (n = 65)	2.11	
Daytime driving	Completely obstructed in at least one eye (n = 59)	2.27	0.116
	Completely or Partially patent (n = 40)	1.95	
Nighttime driving	Completely obstructed in at least one eye (n = 57)	2.49	0.081
	Completely or partially patent (n = 37)	2.00	
Working at a Computer	Completely obstructed in at least one eye (n = 69)	2.70	0.021^*^
	Completely or partially patent (n = 40)	2.13	
Watching TV	Completely obstructed in at least one eye (n = 123)	2.48	0.000^**^
	Completely or partially patent (n = 72)	1.86	
Work-related activities	Completely obstructed in at least one eye (n = 65)	2.49	0.031^*^
	Completely or partially patent (n = 48)	1.98	
Household activities	Completely obstructed in at least one eye (n = 118)	2.19	0.037^*^
	Completely or partially patent (n = 64)	1.77	
Outdoor activities	Completely obstructed in at least one eye (n = 124)	2.98	0.003^*^
	Completely or partially patent (n = 70)	2.47	
Interpersonal relations	Completely obstructed in at least one eye (n = 126)	2.63	0.000^**^
	Completely or partially patent (n = 71)	1.99	
General happiness	Completely obstructed in at least one eye (n = 105)	2.13	0.005^*^
	Completely or partially patent (n = 65)	1.55	

^*^P < 0.05, ^**^P < 0.01.

## Discussion

Ocular surface diseases are relatively common ocular problems. Several forms of evidence have suggested that ocular surface diseases reduce vision-related QOL. In 1992, Reiger [8] reported that a stable pre-corneal tear film was important in optical image quality. However, it is hard to measure discomfort independently and objectively, and self-assessment methods have been used for symptom analysis. Since Reiger’s report, several studies have shown that impaired tear film degraded vision-related QOL [9–11]. For estimating objective discomfort, a generic scale, such as the National Eye Institute Vision Functioning Questionnaire (NEI-VFQ), and a disease specific questionnaire, such as the Impact of Dry Eye on Everyday Life (IDEEL) and the OSDI, have typically been used. For dry eye syndrome, IDEEL [12] and OSDI [13] are typically used. IDEEL comprises three components; i.e., QOL, therapeutic satisfaction, and annoyance of symptoms assessment, and 57 time-consuming questions. The OSDI comprises three sub-measurements; i.e., vision-related functions, eye symptoms, and environmental risk factors, 12 questions and is widely used to assess the discomfort of dry eye syndrome and therapeutic effects. Usually, patients with nasolacrimal duct obstruction are older than patients with dry eye syndrome. Even when given detailed descriptions, the evaluation methods need to be further simplified. The success or failure of dacryocystorhinostomy has been evaluated using a lacrimal irrigation test, measurement of the height of the lacrimal lake, the amount of residual fluorescein in a dye disappearance test, and measurement of the size of the bony opening in an endonasal endoscopy test [14]. However, these objective measurements do not totally represent improvement of discomfort. Cassel [15] also insisted that, not only objective results of medical procedures, but also measurement of the patient’s subjective satisfaction is important. This study used 10 questions to assess vision-related QOL in the epiphora patients with reference to OSDI with simple scoring from 0 to 4. From analysis of the patients who underwent dacryocystorhinostomy, all item scores were significantly decreased postsurgery, which reflected the usefulness of the items for QOL assessment in epiphora patients.

Self-assessment results showed that epiphora most greatly hindered outdoor activities. Reflex tearing from wind and external stimuli may be excessive during such activities. As more people pursue health and leisure activities, outdoor activities are regarded as important in daily life, and epiphora can be an annoying symptom that prevents enjoyment of these activities. Indoor activities such as working at a computer, work-related activities, and interpersonal relations showed relatively high scores. In the age related analysis, older patients tended to have lower scores in interpersonal relations, which might reflect older people receding from active social life and having less discomfort from epiphora in interpersonal relations than younger people. Otherwise, all other item scores showed no age-related correlation.

Female patients had higher scores for household activities, outdoor activities, and interpersonal relations than males. In South Korea, household activities are traditionally performed by women, and discomfort from epiphora during household activities was seen as less of a concern for males. As gender roles in this society change, the impact of epiphora on QOL may evolve to another pattern of gender influence. There was no difference in epiphora's impact on vision-related QOL, based on the bilaterality of epiphora symptoms. This indicated that the symptom of epiphora itself creates significant discomfort in daily life, so there is no QOL difference between one-eye and two-eye epiphora.

Notably, those with complete obstruction of at least one eye in a lacrimal irrigation test showed higher scores for all 10 questions and denoted a statistically significant difference in eight questions. We therefore found that such epiphora creates greater discomfort than partial obstruction or patent lacrimal systems. A completely obstructed lacrimal passage evidently not only hinders lacrimal drainage passage more significantly but also builds up secretion from consequent inflammation [16]. The mucinous secretion in the tear lake from increased goblet cells of the lacrimal sac blurs imaging and lowers the vision-related QOL.

Even though a small number of patients was available for a one-time questionnaire session during the postoperative follow-up period to allow before and after surgery comparisons, dacryocystorhinostomy significantly lowered self-assessment scores for all 10 questions (p < 0.01). In accordance with previously reported articles [4, 5, 7], dacryocystorhinostomy can improve vision-related QOL for epiphora patients.

The 10-item questionnaire was easy to perform and efficiently showed vision-related QOL in epiphora patients. This QOL is hindered in many aspects of daily life, which need to be considered in patient management. Because of the nature of nasolacrimal duct obstruction, uneven gender distribution was inevitable and additional older patients were included in the study. The relatively low response rate for some items was attributed to these two factors. To resolve these problems, we used nonparametric methods in the statistical analysis but some biases might have occurred. We did not perform a study to validate and standardize our questionnaire and we used a modified version of the OSDI, which was another limitation of this study.

## Conclusion

This study first evaluated subjective vision-related discomfort in epiphora patients. Our questionnaire was easy to complete and it efficiently reflected vision-related QOL. QOL in epiphora patients was hindered in many aspects of daily life, which needs to be considered in patient management. We showed that epiphora from the patient’s perspective, can impact QOL. Our results could help to improve overall therapeutic strategies and surgical management of patients with epiphora.
